# Respiratory Chain Complexes in Dynamic Mitochondria Display a Patchy Distribution in Life Cells

**DOI:** 10.1371/journal.pone.0011910

**Published:** 2010-07-30

**Authors:** Britta Muster, Wladislaw Kohl, Ilka Wittig, Valentina Strecker, Friederike Joos, Winfried Haase, Jürgen Bereiter-Hahn, Karin Busch

**Affiliations:** 1 Institute of Kinematic Cell Research, Department of Biology, University of Frankfurt, Frankfurt/Main, Germany; 2 Laboratory for Mitochondrial Dynamics, Department of Biology, University of Osnabrueck, Osnabrueck, Germany; 3 Institute of Molecular Bioenergetics, Medical School, University of Frankfurt, Frankfurt/Main, Germany; 4 Electron Facility, Max Planck Institute of Biophysics, Frankfurt/Main, Germany; Université de Toulouse, France

## Abstract

**Background:**

Mitochondria, the main suppliers of cellular energy, are dynamic organelles that fuse and divide frequently. Constraining these processes impairs mitochondrial is closely linked to certain neurodegenerative diseases. It is proposed that functional mitochondrial dynamics allows the exchange of compounds thereby providing a rescue mechanism.

**Methodology/Principal Findings:**

The question discussed in this paper is whether fusion and fission of mitochondria in different cell lines result in re-localization of respiratory chain (RC) complexes and of the ATP synthase. This was addressed by fusing cells containing mitochondria with respiratory complexes labelled with different fluorescent proteins and resolving their time dependent re-localization in living cells. We found a complete reshuffling of RC complexes throughout the entire chondriome in single HeLa cells within 2–3 h by organelle fusion and fission. Polykaryons of fused cells completely re-mixed their RC complexes in 10–24 h in a progressive way. In contrast to the recently described homogeneous mixing of matrix-targeted proteins or outer membrane proteins, the distribution of RC complexes and ATP synthase in fused hybrid mitochondria, however, was not homogeneous but patterned. Thus, complete equilibration of respiratory chain complexes as integral inner mitochondrial membrane complexes is a slow process compared with matrix proteins probably limited by complete fusion. In co-expressing cells, complex II is more homogenously distributed than complex I and V, resp. Indeed, this result argues for higher mobility and less integration in supercomplexes.

**Conclusion/Significance:**

Our results clearly demonstrate that mitochondrial fusion and fission dynamics favours the re-mixing of all RC complexes within the chondriome. This permanent mixing avoids a static situation with a fixed composition of RC complexes per mitochondrion.

## Introduction

Mitochondria have multiple functions in catabolism, biosynthesis, iron and Ca^2+^ homeostasis, and signaling, but their major function is the synthesis of ATP, the general energy currency of cells. ATP is the final product of the coordinated action of five protein complexes, which are localized in the inner mitochondrial membrane. Whereas complex I (NADH-ubiquinone:oxidoreductase), complex II (succinate dehydrogenase), complex III (cytochrome *c* reductase, the b*c*1-complex), and complex IV (cytochrome *c* oxidase, COX) constitute the redox portion of the RC, Complex V is the F_o_F_1_-ATP synthase [1], [2]. Their concerted action, termed oxidative phosphorylation, couples a series of redox reactions to the generation of a proton motive force across the inner mitochondrial membrane, which then drives ATP synthesis [3]. The redox activity of complexes I and III is also the main source of reactive oxygen species (ROS) [4]. In a vicious cycle, ROS can cause functional damage, i.e. reduced coupling and ever increasing ROS production [5], posing a threat for both the mitochondria and the cell with severe consequences for the proper function of organs and consequently, organisms [6], [7], [8], [9]. It is suggested that mitochondrial dynamics under normal conditions counteracts this problematic scenario [10], [11]. In many cell types mitochondria fuse and divide frequently [12], [13], [14], [15], [16], [17], [18]. The balance between fusion and fission controls mitochondrial morphology and probably function and depends on many variables as are cellular physiological and developmental states [7], [19], [20], [21] and environmental factors [22], [23]. Pathologic or experimentally induced imbalance of fusion and fission correlates with altered mitochondrial ultrastructure, impaired mitochondrial function, loss of mitochondrial DNA, and depolarization of inner mitochondrial membranes [22], [24], [25], [26] and is linked to several neuro-degenerative diseases [12], [27], [28], [29], [30], [31] as well as apoptosis and aging [16], [32], [33], [34], [35], [36], [37]. Several observations support the idea of a rescue function of normal mitochondrial dynamics [38], suggesting a double function in quality control as well as re-mixing of mitochondrial compounds. Accordingly, mitochondrial fission allows the separation and elimination of seriously damaged organelles by autophagy [11], [39], [40], [41], while fusion enables the exchange of mitochondrial compounds. Cells lacking mitochondrial fusion — due to deletion of the fusion proteins Mfn1 and Mfn2 or loss of OPA1— show severe cellular defects [42], including slow growth and reduced activity of all respiratory complexes. The rescue hypothesis suggests that the continuous remixing of mitochondrial compounds due to fusion provides a short-term rescue by re-equilibration of the membrane-potential [39], [40]. Remixing of proteins - especially the respiratory complexes - could prevent the local accumulation of damaged proteins by diluting them with functional ones. Exchange of mtDNA between dynamic mitochondria is known for long time [43], [44], [45], but the time scale of complementation inclusive protein de novo synthesis is different from what we investigated here.

Here we have studied the spatiotemporal distribution of RC complexes and ATP synthase in the context of mitochondrial dynamics in a time frame of several hours. With appropriately labelled RC complexes and ATP synthase we determined their localization before and after mitochondrial fusion.

## Results

### Labelling of respiratory chain complexes by integration of fluorescent subunits

**Figure 1:**
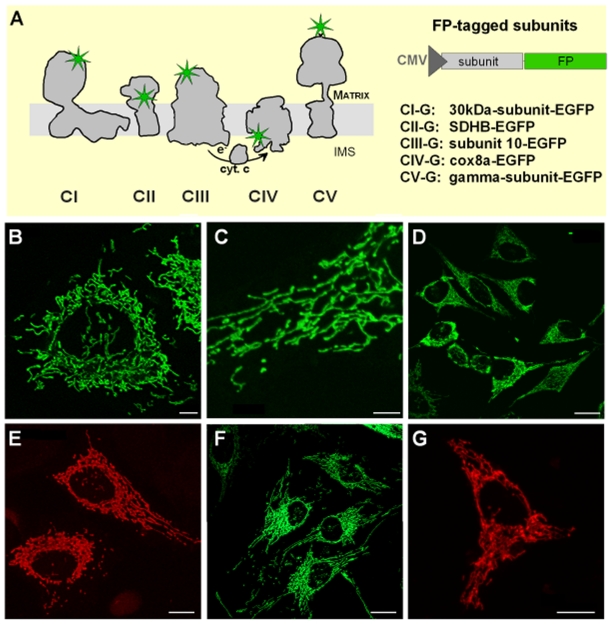
Labelling of RC complexes in human cells by fusion to fluorescent proteins. A. Mitochondrial RC complexes were labelled by fusion of single subunits to monomeric fluorescent proteins mEGFP, mDsRed and mDendra2, resp. at the indicated sites. B.C. Expression of the complex II subunit SDHB fused to GFP (CII-G) and its mitochondrial localization in stably transfected HeLa cells as visualized by fluorescence microscopy. D. Expression of the complex III subunit 10 (6.4 kDa UQCR) fused to GFP (CIII-G) and its mitochondrial localization in stably transfected HeLa cells as visualized by fluorescence microscopy. E. Expression of complex IV subunit 8a fused to hAGT and labelled with BG-TMR in stably transfected HeLa cells (CIV-hAGT-TMR). F. Expression of complex IV subunit 8a fused to GFP in stably transfected HeLa cells (CIV-G). G. Mitochondrial localization of complex V labelled at its γ-subunit by fusion to DsRed in stably transfected HeLa cells (CV-R). Scale bars: 20 µm (D, F, G); 5 µm (B, C), and 10 µm (E).

The complexes of the RC and ATP synthase were labelled by fusion of subunits to monomeric forms of auto-fluorescent proteins FP (EGFP: enhanced green fluorescent protein and DsRed: red fluorescent proteins from *Discosoma* sp.). Alternatively, a hAGT-tag was fused and stained with a fluorescent substrate [46], [47]. Preferentially, subunits of less pronounced functional importance were chosen for labeling. The tagging and choice of subunits were designed in consideration of the known 3D structures of the RC complexes and ATP synthase complexes (*Bos taurus*: Protein data bank codes 1BMF, 2EIN, 1LON, *Gallus gallus*: 2FBW) as well as the existing knowledge of their function. The proteins were tagged at their C-termini to avoid interference with the N-terminal mitochondrial targeting sequences. Complex I was labelled at its 30 kDa subunit and the resulting cell line was termed CI-G when fused to EGFP and CI-R when fused to DsRed. Complex II was tagged at subunit B (CII-G and CII-R, respectively), complex III at subunit 10 (6.4 kDa subunit) (CIII-G and CIII-R, respectively), complex IV at the Cox8a subunit (CIV-G, CIV-R, and CIV-hAGT, respectively) and ATP synthase at the γ-subunit (CV-G and CV-R, respectively) as depicted in Figure 1A. For ATP synthase, the γ-subunit was tagged, which in yeast maintains its functionality if tagged C-terminally [48] as we did here. Figure 1A summarizes the positions of the FP-tagged subunits in the holo-complexes of oxidative phosphorylation.

The tagged subunits were correctly targeted to mitochondria (Figure 1B–G) as visualized by fluorescence imaging of transfected cells. For subsequent experiments, stable cell lines with moderate expression levels of RC complexes and ATP synthase-FP were selected. In the stable transfected cell lines cytosolic localization of FP-subunits was beyond the detection limit (Figure 1B–G).

**Figure 2:**
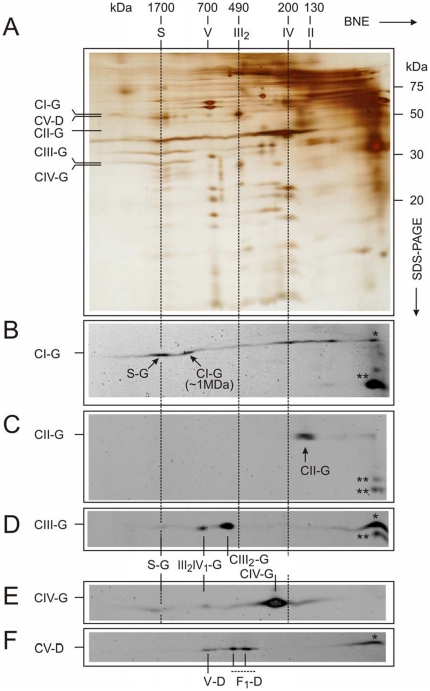
Assembly of fusion proteins into mitochondrial complexes in HeLa cells was tested by in-gel fluorescence after 2-D BN/SDS-PAGE. GFP and Dendra2 fluorescence was preserved after SDS-PAGE and signals are shown in grayscale (lower panels). A. The same gels were silver-stained. The gel of CIV-G expressed HeLa cells was shown as an example. The positions of the fusion proteins were marked (left axis). B. Complex I – 30 kDa-GFP (CI-G) was detected in complex I, in supercomplexes (S–G) and in subcomplexes of complex I. C. Complex II-subunit B-GFP (CII-G) was predominantly found in complex II. D. Complex III-6.4 kDa subunit -GFP (CIII-G) was predominantly found in the dimer of complex III (CIII_2_-G), additionally in small and large supercomplexes (S–G and III_2_IV_1_-G). E. Complex IV-Cox8a-GFP (CIV-G) was detected predominantly in complex IV, and smaller amounts were assembled into supercomplexes (S–G and III_2_IV_1_-G). F. Complex V-γ-Dendra2 (CV-D) was detected in holo complex V (V–D) and, in F_1_ subcomplexes (F_1_-D). At the gel front, individual fusion proteins (*) together with degradation products (**) were detected (B–D, F). S, large supercomplexes composed of respiratory chain complexes I, III_2_, and IV; III_2_IV_1_, small supercomplexes composed of respiratory chain complexes III_2_, and IV; V, complex V or ATP synthase; IV, complex IV or cytochrome c oxidase; III_2_, dimer of complex III or cytochrome c reductase; II, complex II; F_1_, subcomplex of complex V; G, GFP; D, Dendra2, BNE, Blue native electrophoresis.

To prove the assembly of FP-tagged subunits into the holo-complexes of the respiratory chain, we performed 2D Blue native electrophoresis to separate complexes. In the second dimension, a SDS/PAGE was performed to split protein complexes into their subunits and fluorescent fusion proteins were detected by laser scanning. The same 2D-gels were silver stained and the positions of the fusion proteins were mapped (Figure 2A). The gels show that fusion proteins were assembled into the individual holo-complexes and supercomplexes of the respiratory chain (Figure 2 B–F). Complex I –GFP (CI-G) was detected in complex I (1 MDa) as we recently demonstrated [49] but also in supercomplexes (S–G) (Figure 2B). Supercomplexes of various compositions are known for some years [50], [51], [52], [53], consisting of CI/CIII, CI/CIII/CIV and, CIII/CIV, but not of CII. Complex II-subunit B-GFP (CII-G) was found in complex II (Figure 2C). Complex III-6.4 kDa subunit tagged with GFP (CIII-G) was predominantly found in the dimer of complex III but also in supercomplexes and as free CIII-G marked by an asterisk (Figure 2D). Cox8a-GFP (CIV-G) was detected predominantly in complex IV, and smaller amounts were assembled into supercomplexes (Figure 2E). Complex V-γ-Dendra2 (CV-D) was detected in holo complex V, and in F_1_ subcomplexes as well as γ-FP (asterisk in Figure 2F). At the gel front of the first dimension, individual fusion proteins together with degradation products were detected. These products could be the result of proteolytic degradation during sample preparation. Due to the addition of GFP or Dendra2, fluorescent complexes show a mass shift in the native gel compared to endogenous complexes (Figure 2C–F). Since the main part of silver stained complex IV in the gel from expressed CIV-G cells (Figure 2A) was found at the position of the endogenous complex and not at mass shifted fluorescent complex IV, it is obvious that only a minor part of inner membrane mitochondrial complexes are fluorescently tagged due to the moderate expression level of FP-subunits. In summary, the fluorescent fusion subunits were assembled into mitochondrial membrane complexes, but most complexes contain their endogenous unlabelled subunits in the inner membrane. We thus can exclude overexpression derived artefacts.

### Introduction of fluorescent subunits into RC complexes and ATP synthase in mitochondria does not alter function and ultra-structure of mitochondria

**Figure 3:**
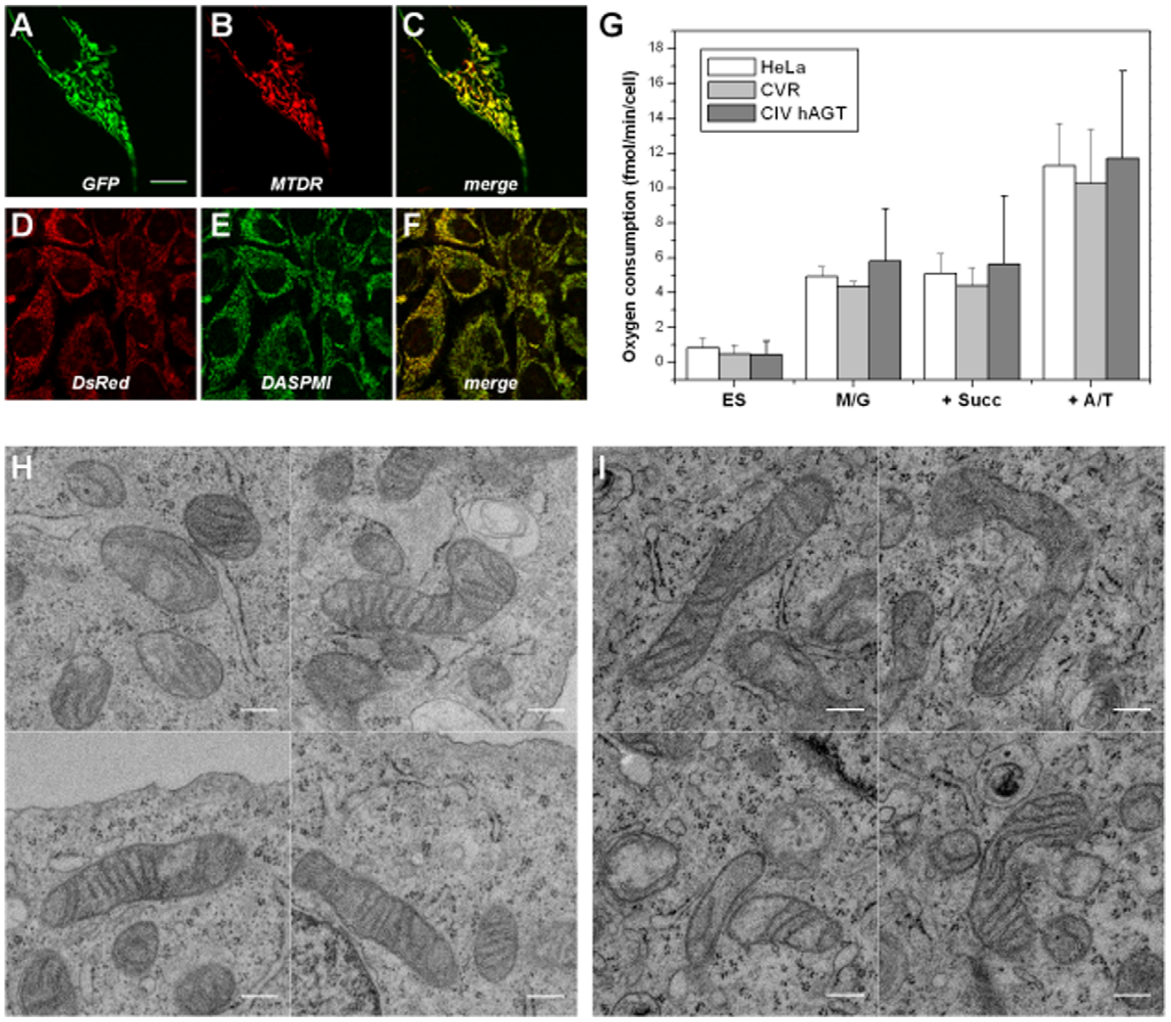
Tagging of subunits of RC complexes does not interfere with mitochondrial localization, membrane-potential generation, respiration, or ultrastructure in stably transfected HeLa cells. A–C. Expresssion of complex III subunit K fused to GFP (CIII-G) and labelling with the mitochondrial-specific dye MitoTracker Deep Red FM. C. The merged image shows clear co-localization indicating correct targeting of complex III subunit 10-GFP to mitochondria. D. The mitochondrial membrane potential in cells stable expressing the modified γ-subunit of complex V (CV-R-cells) is sustained. The merge (F) of the Complex V-γ-DsRed-signal (D) with the DASPMI-signal (E) shows the co-localization of the signal. G. Respiration of CV-R cells and cells stable expressing CIV-hAGT is not altered when endogenous substrate is oxidized, nor when substrates for the different RC complexes are added (complex I is supplied with NADH by the corresponding dehydrogenase feed with malate/glutamate, M/G; complex II is fueled with succinate, succ; and complex IV receives electrons from cytochrome c, which is reduced by TMPD in presence of ascorbate, A/T)[81]. Oxygen consumption rates derive from at n≥3 (CV-R: 4; HeLa: 3; CIV-hAGT: 5) independent cell preparations and measurements. H. Electron micrographs of mitochondria in HeLa cells. I. Electron micrographs of mitochondria in CV-R cells stably expressing the modified γ-subunit. Scale bars: 10 µm (A–C), 20 µm (D–F), 300 nm (H, I).

To test whether the tagging of ATP synthase subunit γ with fluorescent proteins affected its functionality and thus the energy supply of these cells, we analyzed the membrane-potential, and compared growth rates, and respiratory activity of transfected and non-transfected cells. Cytochrome c oxidase was tagged with hAGT (CIV-hAGT) at its subunit 8a. Cytochrome c oxidase is the final enzyme of the respiratory chain. Neither tagging of complex IV nor complex V interfered with the generation of a protonmotive force in mitochondria. Mitochondrial membrane potential – as recorded by DASPMI staining [54], [55] - remained unaltered in cells expressing the γ-DsRed fusion subunit of ATP synthase (Figure 3A–F). CIV-hAGT cells could be successfully stained with MitoTracker DeepRed indicating the presence of a membrane potential, but mitochondria lost this dye after dissipation of the membrane potential by CCCP poisoning (data not shown). Oxygen consumption rates with different substrates were not altered in stable transfected CVR and CIV-hAGT cells in comparison to non-transfected cells (Figure 3G), indicating that the expression and assembly of tagged subunits did not interfere with energy metabolism. Cell proliferation might be a more general but also more sensitive test for physiological functionality. Therefore cell doubling times were recorded over at least 10 days. No significant changes of generation times of CV-R and CIV-hAGT cells in comparison to control were found: The doubling times were 24±0.2 h for non-transfected HeLa cells, 24.5±0.1 h for CV-R and 25.5±0.1 h for CIV-hAGT.

Since ATP synthase is crucial for the formation of mitochondrial cristae [56], [57], we searched for ultrastructural changes in cells expressing CV-R or CIV-hAGT on top of endogenous unlabelled complex. No ultrastructural changes such as differences in shape or size of submitochondrial structures (e.g. cristae) were found in electron micrographs or by fluorescence microscopy of mitochondria in CV-R transfected cells (Figure 3H and I), nor CIV-hAGT cells (not shown). These results support that the moderate tagging of RC complexes and ATP synthase neither affected mitochondrial morphology and ultrastructure, nor- most importantly -their function.

### Spreading of RC within a single cell

**Figure 4:**
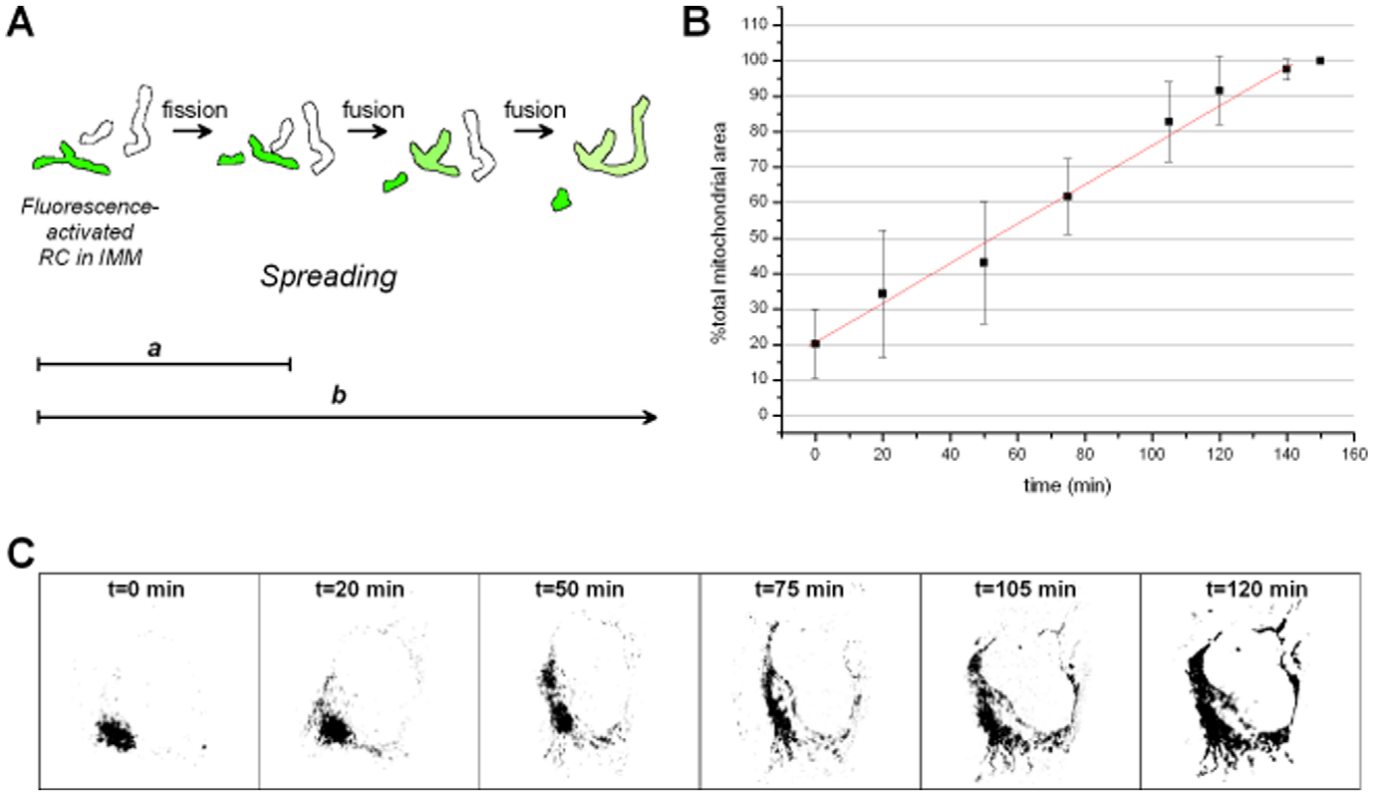
Spreading of RC in single cells due to mitochondrial fusion and fission dynamics and motility. The re-mixing of RC-complexes in intact living cells was shown by using photoactivatable PAGFP fused to complex I. A. Scheme of events leading to a re-mixing of RC. A single event already spreads the signal due to mitochondrial fission dynamics (a), later mitochondrial fusion and fission dynamics overlay with mitochondrial motility distributing the RC further (b). B. Quantification of CI-PAGFP re-location in single cells (n = 7) due to mitochondrial fusion and fission dynamics and motility. C. Time-course of CI-PAGFP spreading in a single cell after area-restricted visualization of CI-PAGFP by photoactivation. After recording a z-t-series, a maximum intensity image from each z-stack was generated and converted into a binary image after background substraction. For better contrast, the images are depicted in the inverted mode.

We first asked how fast mitochondrial fusion and fission dynamics in combination with mitochondrial motility would promote the re-mixing of RC complexes. The use of photoactivatable fluorescent proteins is a versatile tool to monitor mitochondrial dynamics [58], [59]. Figure 4A gives a schematic overview on the contributing processes. Labelling of a RC complex in the IMM with UV-photoactivatable green fluorescent PAGFP enabled the tracking of a certain subpopulation in the chondriome over time. We tagged complex I with PAGFP, generated stable cell lines and monitored the spreading of complex I from about 20% of total mitochondrial volume throughout the chondriome. In average, it took 150 min to disseminate CI-PAGFP within the chondriome (Figure 4B). For demonstration, spreading of CI-PAGFP from on subset of mitochondria is depicted in Figure 4C. For better contrast, the fluorescent mitochondria are depicted in black (inverse mode).

### Fusion and fission result in the exchange of RC complexes and ATP synthase between mitochondria

The RC-PAGFP dispersion assay only reveals the propagation of one RC complex. We next asked to which extend two different RC complexes will be re-mixed in dynamic mitochondria. Exchange and re-mixing of two RC complexes has been monitored by a cell fusion assay: Stable transfected cells with differently fluorescing RC complexes were fused and fluorescence distribution was monitored over a time period from 4–24 h after fusion by fixing cells at different time points for analysis (Figure S1). Subsequent to cell fusion, mitochondrial fusion and fission dynamics generated hybrid mitochondria containing the differently labelled RC complexes and ATP synthase (Figure S1B, C). Successful fusion of the inner mitochondrial membranes requires an intact membrane potential [26], indicating that the activity of RC complexes and ATP synthase should be maintained under these conditions. Even in polykaryons consisting of 5–6 cells (Figure S1C), already 2 h after cell fusion hybrid mitochondria containing both RC-FP complexes were found. This time course corresponds to earlier observations on dynamic mitochondria with matrix targeted FPs (56). 4–5 h after cell fusion, cells were fixed and analysed for the spatial distribution of RC complexes. For measurements longer than 4 h after the onset of cell fusion, cycloheximide (CHX) was added to cell cultures to inhibit protein synthesis and thus exclude incorporation of newly synthesized proteins into the mitochondria. Cycloheximide application directly before or after onset of fusion impaired the recovery of cells and mitochondria, which were stressed by the PEG treatment.

After 24 h the hybridisation of the chondriome and the rearrangements of RC complexes and ATP synthase in polykaryons were completed. Beyond 26 h, the presence of cycloheximide prevented survival of the fused cells due to inhibition of protein synthesis. For comparison, cells were co-transfected with two plasmids (Figure S1D–E) to generate a balanced distribution of differently tagged fluorescent subunits of RC complexes and ATP synthase.

### Recently fused mitochondria appear patchy

**Figure 5:**
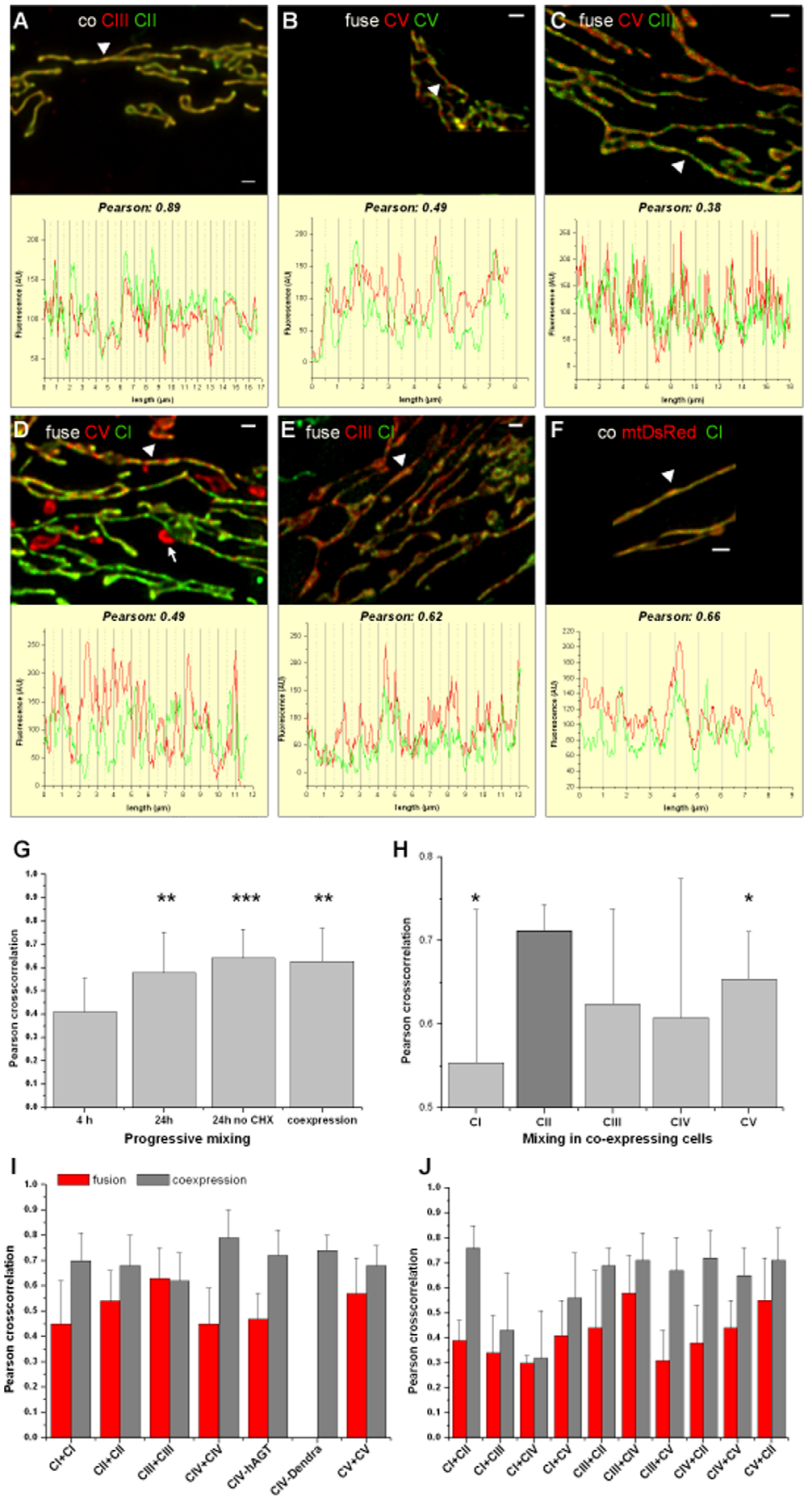
Hybrid mitochondria following mitochondrial fusion display a patterned distribution of RC complexes. Ongoing fusion and fission of mitochondria subsequent to cell fusion caused the generation of hybrid mitochondria with a mixed population of RC complexes. A. Appearance of mitochondria with CIII-R and CII-g 5d after coexpression. Lower part: Line plot of CIII-R and CII-G fluorescence intensities along the longitudinal axis of a single mitochondrion, indicated with a white arrow head in A. The corresponding Pearson's cross correlation coefficient is 0.89 calculated from the fluorescence distribution in a single mitochondrion as depicted in the lower part. B. CV-R and CV-G distribution in hybrid mitochondria 5 h after cell fusion. The corresponding Pearson coefficient is 0.49 calculated from the line plot in the lower part. C. CV-R and CIII-G fusion and analysis 4 h later. Lower part: Line plot of CIII-G and CV-R fluorescence intensities along the longitudinal axis of a single mitochondrion, indicated with a white arrow head in C. The corresponding Pearson's cross correlation coefficient is 0.38. D. CI-G and CV-R in mitochondria 4.5 h after cell fusion. Lower part: Line plot of CI-G and CV-R fluorescence intensities along the longitudinal axis of a single mitochondrion, indicated with a white arrow. The corresponding Pearson's cross correlation coefficient is 0.49. Some fragmented mitochondria display only CV-R fluorescence, because they did not fuse with CI-G-mitochondria (arrow). E. CI-G and CIII-R fluorescence distribution in mitochondria 5 h after fusion. The calculated Pearson for the correlation of the fluorescence distribution is 0.62. F. CI-G and mtDsRed fluorescence distribution in mitochondria in cells co-expressing both plasmids. The corresponding Pearson's cross correlation coefficient is 0.66. Scales bars 1 µm  = 30−40 pixel (A, C, D E), 1 µm  = 108 pixel (B), 1 µm  = 134 pixel (F). After recording z-stacks, the images were deconvolved by ®Autoquant software before quantitative analysis. G. Comparison of fluorescence distribution in CI-G and CV-R fused and co-expressing cells under different conditions reveal an increase in homogeneity by time due to ongoing fusion dynamics. 24 h after cell fusion the mixing of RC is complete as proved by the comparison with co-expressing cells (n≥3 independent cell assays for 4 h, 24 h CHX and coexpression; n = 2 cell assay for 23 h no CHX). H. Complex II mixes better with other complexes in co-expressing cells than complex I and V, resp**. (**students t-test, significance level p = 0.05) I.J. Cells with red and green fluorescent RC complexes were fused and fluorescence distribution of RC complexes in hybrid mitochondria 4–5 h after fusion as well as in mitochondria from co-expressing cells was determined. The cross-correlation of the two fluorescence channels, calculated according to Pearson, was taken as a measure for the homogeneity of RC complex distribution. For each combination at least 15 heterokaryons from 3 different independent cell fusion/co-expression assays were investigated. The data are shown as mean ± s.e.m (n≥3). Significance of differences from the relevant controls was calculated by Students' *t* test.

From studies with matrix-proteins it is known that these spread fast and complete in fused mitochondria, while IMM proteins mix slower [60]. Only a complete rearrangement of the inner membrane and unlimited lateral mobility of RC complexes and ATP synthase-complexes within it should result in a homogeneous distribution of both fluorescences across the hybrid mitochondria, whereas the maintenance of cristae and restricted diffusion would prevent total and thus homogenous mixing. Indeed, volume images of hybrid mitochondria displayed a more patterned distribution of the differently coloured RC complexes and ATP synthase in fused cells (Figure 5B–E) than in cells co-expressing two complexes. Along the axis of single mitochondria the red and green fluorescence from differently labelled complexes alternated. For quantification, the fluorescence of a GFP-tagged complex was depicted separately from the fluorescence of a RFP-tagged complex, and Pearson's cross-correlation parameter was then determined (see M&M). The higher the Pearson's cross-correlation parameter (*r*_P_), the higher is the degree of co-localization. The squared value of *r*_P_ corresponds to the percentage of the variance of the green channel, which can be explained by the changes of the red channel and vice versa, thus for *r*_p_  = 0.66, its squared value (0.66^2^ = 0.43), 43% of the fluorescence variances correspond to each other. The rest (100−43 = 57%) of the variances is “independent” of the other channel. Different combinations were tested to check whether different complexes would mix differently. CI/CIII and CIV have been expected to be at least partially assembled in macrocomplexes and for CV dimerization and on top arrangement in rows is well known. In contrast, complex II was regularly not found in supercomplexes (61). We suspected a difference in mixing if macromolecular supercomplexes would have influence on the mobility and local arrangement of RC complexes. Figure 5 compares four different fusions (CI-G and CIII-R, CII-G and CV-R, CIII-G and CV-R, CV-R and CV-G), and the distribution of RC in co-expressing cells. As an additional control, the distributions of CI-G and mtRed were depicted and analysed in a co-expressing cell.

Figure 5A shows the fluorescence image of few mitochondria from a cell co-expressing complex III tagged at 6.4 kDA-subunit with DsRed and complex II subunit SDHB tagged with EGFP, resp. (co CIII-R and CII-G). The correlation of red and green fluorescence distrubtion along a single mitochondrion (indicated with an arrow head) yielded a Pearson's coefficient of 0.89 (Figure 5A). In Figure 5B, fusion of cells expressing either CV-G or CV-R ended up with patchy hybrid mitochondria and a Pearson cross-correlation coefficient of 0.49 (Figure 5B).

Figure 5C shows the hybrid mitochondria after fusion of CV-R and CIII-G and the fluorescence distribution µm along the long axis of a single mitochondrion. The resulting Pearson's coefficient was 0.38. The same was performed for CI-G and CV-R 4 h after fusion, yielding a specific Pearson of 0.49 (Figure 5D). The arrow indicated a non-fused mitochondrion with only CV-R fluorescence. For CIII-R and CI-G a Pearson of 0.62 in the arrow-head indicated mitochondrion was determined (Figure 5E). In comparison, coexpression of complex I 30 kDa-GFP (CI-G) with matrix-targeted DsRed (mtRed) yielded an intermediate Pearson coefficient (*r*_P_ = 0.66; Figure 5F). The CI-G fluorescence distribution in comparison to the mtDsRed (matrix located) fluorescence distribution is more irregular than in cells that co-express two membrane-associated RC complexes or ATP synthase. This reflects the different submitochondrial localization of CI-G as an inner membrane protein and mtDsRed as a matrix-targeted protein.

In a time dependent assay we then investigated the mixing of CI-G and CV-R. CI-G and CV-R together are usually not found in larger macromolecular supercomplexes, so we expected an independent mixing. Four hours after induced cell fusion the *r*_P_ in fused hybrid mitochondria was 0.42±0.15. The Pearson value then further increased due to ongoing fusion and fission. 24 h after fusion, the re-mixing of CI-G and CV-R was complete, independent from the number of contributing cells (usually 4–6) in the polykaryon. The Pearson reached the value of RC distribution in coexpressing cells (Figure 5G). To test whether cycloheximide in the fusion culture had an influence on the mixing, cycloheximide was omitted in parallel cultures, but no significant difference in *r*_P_ for RC complexes and ATP synthase distribution was observed 24 h after fusion in cultures with and without cyclohemixide.

We next asked whether the distribution pattern was dependent on the respective RC complexes and ATP synthase combination in the experiment. It is conceivable that complexes usually found in supercomplexes like CI, CII and CIV would display a different mixing behavior then CII or CV, which are not part of these macromolecular arrangements. To complete the analysis, all possible red-green labelled RC complex pairs were investigated. First, the patterned distribution of RC complexes and ATP synthase proved to be reproducible in all recently fused mitochondria (Figure 5I and J, fusion). Due to the rather high sample standard deviations, a distinct mixing behaviour of different RC complexes could not be deduced with certainty. This variance has several causes: i) depending on the position in the polykaryon the hybrid mitochondria underwent already more or less fusion/fission cycles, and ii) the number of cells participating in the formation of the polykaryon has some influence on the hybridisation and mixing speed. So, a standardisation is rather difficult for this kind of experiment. For each combination of DsRed and EGFP-labelled RC complexes, at least 3 independent PEG-induced fusion or co-expression assays were performed. In hetero-combinations such as CI and CIII both possible fusion/coexpression combinations, namely CI-G x CIII-R and CI-R x CIII-G were performed. In every experiment, 4–5 polykaryons were examined and at least 5 single mitochondria underwent co-localization analysis.

Second, the observation that the distribution of RC complexes was more homogeneous in cells coexpressing two fluorescent complexes than in fused hybrid mitochondria 4 h after cell fusion was confirmed for nearly all combinations (Figure 5I and J, coexpression). Exceptional is CIII where the mixing after fusion was rather high, resembling the distribution in co-expressing cells. This could be explained, if CIII-6.4 kDa subunit was not completely integrated in complex III as the BNE analysis suggests. Free CIII-6.4 kDa subunit, assumably in the matrix, would equilibrate in addition by diffusion through the matrix-space and therefore increase the mixing. In contrast, the difference in homogeneity of CII distribution together with CI, CIII and CIV between fused and co-expressing cells was significant. Noticable, CI, CIII and CIV form respiratory supercomplexes, while CV and CII do not participate. Could a microcompartmentation – such as the formation of respiratory strings – be the reason for this reduced mixing?

On average, the Pearson's coefficient for the co-localisation in co-expressing cells was 0.63±0.13. Complex II in co-expressing cells was more homogeneously distributed than complex I and V, resp. (mean Pearson 0.71±0.03, significance p = 0.05) (Fig. 5H). Indeed, this result argues for higher mobility and less integration of complex II in supercomplexes.

As a control, CIV was fused to Dendra2, which is a photoconvertible fluorescence protein. By UV irradiation the green fluorescence was switched to a red emitting species [61]. This was performed to achieve a random mixture of green and red CIV-Dendra2. Since molecules switch stochastically, an unbiased distribution of red and green CIV-Dendra2 and a maximum of co-localisation was expected. The corresponding *r*_P_ was 0.74±0.06 and thus the same as for CIV-G and CIV-R co-expression (*r*_P_ = 0.79±0.11). To check whether the fluorescence pattern might be caused by the use of fluorescent fusion proteins, a CIV-hAGT construct was used [62]. Transfected cells were labelled with different organic dyes instead of fluorescent proteins, fused and analysed. No difference in the *r*_P_ was found in comparison with CIV-G and CIV-R fusion and co-expression systems. The observed fluorescence distribution patterns were also reproducible and independent of the microscope setups.

## Discussion

Mitochondrial dynamics - due to the high rate of mitochondrial fusion and fission [21], [40] and motility [63] - causes equilibration of matrix-targeted proteins [64], [65] within hours throughout the chondriome, but no quantitative data for the concurrent exchange of IMM RC complexes and ATP synthase have been published. Mitochondrial fusion and fission result in the re-distribution of RC complexes and ATP synthase in between mitochondria. During this process hybrid mitochondria with mixed composition are formed. This steady re-mixing of RC complexes and ATP synthase is of particular interest with respect to a putative rescue mechanism in the context of mitochondrial quality control. Mitochondrial RC complexes and ATP synthase- are the main suppliers of reactive oxygen species and also their immediate targets. During their life span, oxidative stress and functional impairment of mitochondria is a prime factor for aging, cancer and many other diseases [35], [66], [67]. Re-mixing could avoid local accumulation of oxidatively impaired proteins and thus rapidly improve overall performance of a mitochondrion. Here, we addressed this important question by recording the re-mixing of respiratory chain complexes and ATP synthase in the context of mitochondrial dynamics. Spreading of a cyan-photoactivated, greenfluorescent tagged RC within the chondriome of a single cell demonstrated the mobility and exchange of IM RC-complexes. Starting this process from a subset of 20% of mitochondrial volume throughout the entire chondriome took about 2–3 h to reach equilibration. This is somewhat slower then the spreading of matrix-targeted PAGFP, which showed an average equilibration time of about 45 min in INS1 cells [68]. This is in line with the observation that matrix-targeted proteins are exchanged also during transient fusion, while IM proteins are much slower spread [60].

Using the PEG-fusion assay, we found a continuous re-mixing of labelled RC complexes and ATP synthase in a syncytium of fused cells. Within 10–24 h, the chondriome derived from 4–6 fused cells was re-mixed completely and all mitochondria had turned into hybrids with respect to their RC complexes and ATP synthase content. This is similar to observations from different groups reporting the complete mixing of mitochondrial content after PEG-induced cell fusion in 7–24 h [64], [69], [70] Apparently, individual mitochondria do not exist for prolonged time spans. The mixing of RC complexes and ATP synthase due to fusion and fission was not homogeneous, though. The resulting hybrid mitochondria display an alternating pattern of fluorescence, indicating incomplete mixing across the inner mitochondrial membrane. This is strengthened by the observations that also xeno-cybrid cells from mouse and human display hybrid patterned mitochondria with respect to the RC complex distribution (data not shown). 24 h after fusion, the fused cells displayed the same respiratory complex distribution as co-expressing cells, underlining that mixing of RC-complexes due to mitochondrial dynamics is a progressing process.

The tagged molecules become incorporated within the RC and ATP synthase, respectively, and part of the complexes then participate in the formation of supercomplexes as was shown by BN-PAGE (Fig. 3). Nevertheless, we cannot exclude that some of the tagged proteins do not integrate into functional complexes, e.g. CIII- 6.4 kDa subunit, and not all of the complexes take part in formation of supercomplexes and thus remain more mobile within the hybrid organelles. The co-existence of tagged proteins with different diffusibility is revealed by different fluorescence patterns: Even in cells co-expressing the same protein with different tags never full co-distribution was reached. In fused (and co-expressing) cells a mobile fraction mixes readily (such as CIII-FP nicely demonstrates) and only the immobile fraction contributes to the patchy appearance. Thus, neither full co-distribution nor full separation of the tags can be achieved. An additional factor is the limitation of microscopic resolution which does not allow the identification of substructures of the IMM such as single cristae. Complete equilibration of matrix proteins is in favour of one mobile fraction only [59], [71] and a homogenous matrix space, and argues against the model of mitoplast organization after fusion as found after drug intoxication [73], [81].

**Figure 6:**
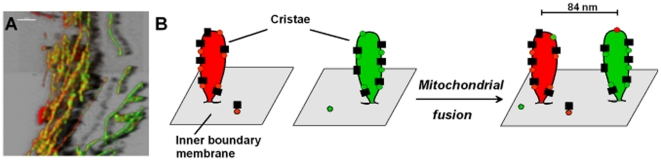
Model of putative cristae preservation during mitochondrial fusion. A. Hybrid mitochondria with patterned distribution of CI-G and CV-R 4.5 h after cell fusion. B. Possible arrangement of cristae in recently fused mitochondria explaining the pattern of hybrid mitochondria. In HeLa mitochondria with regularly arranged cristae, the mean distance between cristae is 84±16 nm.

We interpret the patterned arrangement of RC complexes and ATP synthase as the result of their integration in supercomplexes within cristae. Although cristae rearrangements are known [72], [73], our observations would rather support a model that after organelle fusion at least parts of inner membrane structures are maintained (Figure 6). If cristae are a direct product of inner membrane fusion following outer membrane fusion under control of the fusion proteins Mgm1/Opa1 [74] as it was recently suggested [75], indeed mixed cristae would result at these fusion sites. However, electron micrographs at recent fusion sites suggest that these are rather cristae poor [49]. From our results we cannot exclude, though that a limited subpopulation of complexes might be more mobile and cross cristae borders. Clearly, further studies are needed to reveal the mode of inner membrane fusion and exchange of respiratory complexes in detail on the structural and molecular level.

## Materials and Methods

### Plasmid constructs

Plasmid cassettes with different fluorescent fusion proteins were generated by substitution of hAGT into the pSEMS-26 m vector from @Covalys. Monomeric EGFP was created by introducing an altered codon A206K [76] into the EGFP sequence of the ®Clontech vector pEGFP-N1 (gift from J. Sieber, Göttingen), and monomeric DsRed was inserted after amplification of DsRed from the pDsRed-Monomer-N1 vector from ®BD biosciences. CIV-hAGT is a commercially available vector (pSEMS1-Cox8A-26 m from ®Covalys). A modular system was generated where N-terminally the RC complexes and ATP synthase and C-terminally the fluorescent proteins mEGFP, mDsRed, Dendra2 or hAGT were exchangeable. Thus we could easily generate expression vectors for all combinations of RC complexes and ATP synthase- with all fluorescent proteins (pSEMS-26 m vector derivatives).

Complex I was labelled at its 30 kDa subunit as described earlier [49]. The ORFs of ubunit SDHB from complex II, subunit 10 (6.4 kDa UQCR) from complex III, the cox8a subunit from complex IV and the γ-subunit from ATP synthase were amplified from a self made cDNA library from HeLa cells with the according primers to insert in the multiple cloning sites of the pSEMS-26 m vector derivatives.

### Cell culture

HeLa Cells were grown in MEM's Earle's with stable Glutamine (Biochrom AG, FG0325) medium supplied with 10% foetal bovine serum superior(FBS) (Biochrom AG, S0615), 1% MEM non essential amino acids (PAA laboratories GmbH M11003) and 1% 2-(4-(2-Hydroxyethyl)- 1-piperazinyl)-ethansulfonacid HEPES buffer at 37°C with 5% CO_2_. 3T3 cells were grown in DMEM with 10% FBS superior (Biochrom AG, S0615), 3% Glutamin (Biochrom AG, K0302) and 1% HEPES buffer at 37°C with 5% CO_2_.

For the generation of stable cell lines, cells transfected by the calcium phosphate method [77] were selected for stable neomycine resistance by growing in the presence of 0.8 µg/ml G418 (Calbiochem 345810). For our experiments, cell clones with homogeneous and moderate expression of labelled subunits were chosen and proliferated. The arrangement of the fusion protein and the resistance gene under one promoter facilitated the successful selection.

For the fusion assays, differently labelled cells were co-seeded and grown until cell density was between 80–90%. Fusion was initiated by addition of 40% PEG (1500) in PBS for 50 sec, followed by multiple washing steps with PBS and further incubation in medium at 37°C for the indicated time periods. After certain time points after fusion, cells were fixed with 4% paraformaldehyde in PBS for 20 min and for imaging mounted in PBS.

### Electrophoresis and in-gel fluorescence detection

1-D BNE (blue native electrophoresis), and 2-D SDS-PAGE was performed as described using digitonin for solubilization of sedimented cells [78], [79]. Briefly, sedimented cells (HeLa cells and embryonic chicken fibroblasts) were homogenized in diluted sucrose buffer (83 mM sucrose, 6.6 mM imidazole/HCl, pH 7.0) using a motor-driven, tightly fitting 2 ml glass/Teflon Potter-Elvehjem homogenizer. Aliquots corresponding to 20 mg of cells were centrifuged for 10 min at 20,000 g to obtain sediments containing nuclei, mitochondria and larger cell fragments. Pellets were solubilized in 35 µl solubilization buffer (50 mM NaCl, 2 mM 6-aminohexanoic acid, 1 mM EDTA, 50 mM imidazole/HCl, pH 7.0) and 10 µl 20% digitonin. Following 20 min centrifugation at 20,000×g, the supernatant was supplemented with 2.5 µl of a 5% Coomassie blue G-250 suspension in 500 mM 6-aminohexanoic acid. 20 µl of each sample, corresponding to 10 mg cell sediments, was applied to the gel wells (0.15×0.5 cm). Following BNE, gel strips were placed on a glass plate, wetted with 1% SDS for 10 min, and a Tricine–SDS-gel was cast for second dimension. 2-D SDS-gels were scanned using a Typhoon scanner (GE Healthcare) to detect fluorescence of RFP (exitation 533 nm, emission filter 580 nm) and of mCherry (exitation 533 nm, emission filter 610 nm). The same gel was silver-stained [80].

### Fluorescence Microscopy

Co-localisation experiments with fused or co-expressing cells fixed after certain time points were followed using the Leica SP5 confocal microscope equipped with a 63×1.3 APO objective, some with a Zeiss Meta 510 system (63×1.4 NA objective). Fluorescence for red and green fusion-proteins was recorded in a sequential mode to avoid cross-talk. z-stacks of mitochondria were taken in certain region of interests (12×12 µm^2^), with a pixel size corresponding to 30 nm. This is an oversampling of 6 taking into account the Abbé-limit of resolution of 200 nm in y–x. The data-sets were deconvolved using ®Autoquant software to reduce noise and improve image quality.

### Life-cell microscopic imaging

Spreading of RC labelled with paGFP was monitored using an Olympus Fluoview FV1000 cLSM using a 60× objective (UPLSAPO oil, N.A. 1.35) recording 800×800 pixel images as combination of z-stacks and time-series. A Diode laser with 405 nm was used for photoactivation of paGFP in a certain region of interest. A multiline Argon laser was used for imaging paGFP at 488 nm excitation. Spontaneous photoactivation was corrected by background substraction. A Diode laser was used to image MitoTracker-DeepRed at 635 nm excitation. Measurements were performed at 37°C in a self-made Pelletier-heated chamber inclusive objective heating. Images were processed with ®Bitplane Imaris and ImageJ® (NIH, Bethesda, MD, USA).

### DASPMI staining

Cells grown on glass coverslips were stained for 30 min at 37°C with 0.5 µM of the membrane-potential sensitive dye 2-(4-(dimethylamino)styryl)-1-methylpyridiniumiodide DASPMI. Medium was exchanged twice before fluorescence measurements. DASPMI was excited with laser line 470 nm and emission between 560 and 570 nm was recorded.

### MitoTracker DeepRed staining

MitoTracker® Deep Red FM (invitrogen) is a far red-fluorescent dye (abs/em ∼640/662 nm) that stains mitochondria in live cells and its accumulation is dependent upon membrane potential. The dye is well-retained after aldehyde fixation. Cells grown on glass coverslips were stained for 30 min at 37°C with 0.5 µM MitoTracker Deep Red FM. Medium was exchanged twice before fluorescence measurements. DeepRed was excited with laser line 635 nm and emission between 650 and 700 nm was recorded.

### Oxygen consumption measurements

Oxygen consumption measurements were performed as described earlier [81]. In short, a Clark electrode in a waterjacketed chamber connected to a circulating water bath was used. The chamber was sealed with a stopper containing a capillary port for additions. The internal incubation volume was 1 ml. Before measurements, a calibration procedure with sodium dithionite as a reductive was performed. 5−6×10^−6^ cells were harvested by trypsination and permeabilized by addition of 0.75 µl 10% digitonin per 1 ml cell suspension containing 5−6×10^−6^ cells for accessibility of substrates and inhibitors of respiration. To apply substrates into the chamber, appropriate Hamilton syringes fitted with 2.5 inch long needle were used. Respiration medium, freshly prepared, contained 20 mM HEPES (adjusted to pH 7.1 with NaOH), 250 µM sucrose, 10 mM MgCl_2_, 1 mM ADP and 2 mM potassium phosphate, and 10% (w/v) BSA. End concentrations of substrates were: Malate and glutamate 5 mM each; 5 mM succinate; Ascorbate (10 mM) and TMPD (200 µM).

### Electron microscopy

Sample preparation and electron microscopy was performed as described earlier [21]. In short, cultures were fixed using 2.5% glutaraldehyde, postfixed with 1% OsO_4_, scraped cells in 2% agar embedded, cubed block stained with 1% uranyl acetate, dehydrated in a graded series of ethanol, embedded in LV Resin (Agar Scientfic) and polymerized for 16 h at 65°C. Ultra-thin sections mounted on 100-mesh nickel grids supported by formvar film and stained with 2% uranyl acetate for 2 min and Reynolds' lead citrate for 1 min at R.T.

### Colocalization analysis

For co-localisation determination, the fluorescence intensities of DsRed and EGFP-labelled complexes in cells 4–5 h fixed after fusion were plotted separately in the long-section of single mitochondria. For this, each channel image was displayed separately. A free line was drawn along single mitochondria and the line plot analysis function from ImageJ was used to plot the fluorescence intensity value for each pixel of this line. The same was done for the other channel. Then, the correlation coefficient was calculated. For this, Pearson's coefficient was chosen, since it is independent of the overall intensity values, allowing the comparison also of strong and weak fluorescent signals.

### Statistics

For each combination of DsRed and EGFP-labelled RC complexes, at least 3 or more independent PEG-induced fusion or co-expression assays were performed. So, the data are shown as mean ± s.e.m (n≥3). Significance of differences from the relevant controls was calculated by Students' *t* test. In hetero-combinations such as CI and CIII both possible fusion/coexpression combinations, namely CI-G x CIII-R and CI-R x CIII-G were performed. In every experiment/assay, at least 5 polykaryons were examined and at least 5 single mitochondria underwent co-localization analysis.

## Supporting Information

Figure S1 Assays to investigate mitochondrial fusion and fission and exchange of RC complexes. A–C. Formation of hybrid mitochondria in syncytia by PEG-induced cell fusion, A. Scheme of the procedure: Cells expressing differently labelled RC-complexes were co-plated, and treated with PEG to generate a syncytium with a joined cell body (heterokaryon). B. HeLa cells expressing either CI-G or CI-R were fused. Non-fused and fused cells with a mixture of red and green mitochondria are visible (arrows). C. Image of a syncytium 2 h after fusion of cells expressing CIV-G and CV-R. D. Generation of co-transfected cells by introducing two different plasmids. E. Cells, stable expressing CV-R were in addition tranfected with CVG. The greenish-yellowish cells co-express CV-R and CV-G, while the red cells only express CV-R. F. Single cell co-expressing CI-R and CIII-G 3 days after transfection. Scale bars 30 µM (B, E) and 10 µM (C,F).
(0.72 MB TIF)
