# Supplementary figures and images for: Respiratory Chain Complexes in Dynamic Mitochondria Display a Patchy Distribution in Life Cells
Source: PLoS One. 2010 Jul 30;5(7):e11910. doi: 10.1371/journal.pone.0011910 (PMC2912852; doi:10.1371/journal.pone.0011910)

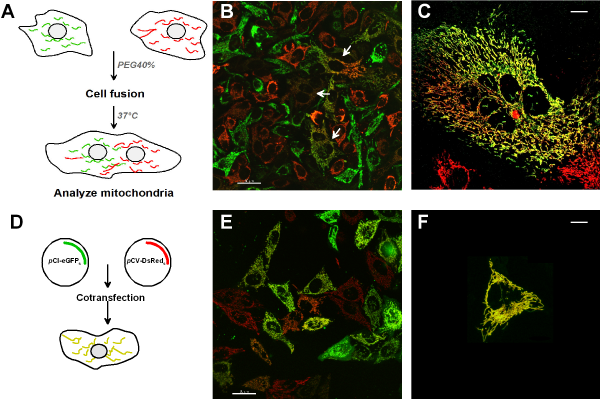

Supplement: Figure S1 — Assays to investigate mitochondrial fusion and fission and exchange of RC complexes. A–C. Formation of hybrid mitochondria in syncytia by PEG-induced cell fusion, A. Scheme of the procedure: Cells expressing differently labelled RC-complexes were co-plated, and treated with PEG to generate a syncytium with a joined cell body (heterokaryon). B. HeLa cells expressing either CI-G or CI-R were fused. Non-fused and fused cells with a mixture of red and green mitochondria are visible (arrows). C. Image of a syncytium 2 h after fusion of cells expressing CIV-G and CV-R. D. Generation of co-transfected cells by introducing two different plasmids. E. Cells, stable expressing CV-R were in addition tranfected with CVG. The greenish-yellowish cells co-express CV-R and CV-G, while the red cells only express CV-R. F. Single cell co-expressing CI-R and CIII-G 3 days after transfection. Scale bars 30 µM (B, E) and 10 µM (C,F). (0.72 MB TIF) [file pone.0011910.s001.tif]
